# ESUR consensus MRI for endometriosis: protocol, lexicon, and compartment-based analysis

**DOI:** 10.1007/s00330-025-11611-3

**Published:** 2025-05-27

**Authors:** Isabelle Thomassin-Naggara, Miriam Dolciami, Luciana P. Chamie, Adalgisa Guerra, Nishat Bharwani, Sue Freeman, Pascal Rousset, Lucia Manganaro, the ESUR endometriosis working group, Miriam Dolciami, Luciana P. Chamie, Nishat Bharwani, Sue Freeman, Pascal Rousset, Lucia Manganaro, Adalgisa Guerra, Giacomo Avesani, Marc Bazot, Teresa Margarida Cunha, Paolo Niccolò Franco, Rosemarie Forstner, Benedetta Gui, Edith Kermarrec, Stefania Rizzo, Hilal Sahin, Shiwa Mansournia, Isabelle Thomassin-Naggara, Laura Buñesch Villalba, Ramona Woitek

**Affiliations:** 1 Imageries Radiologiques et Interventionnelles Spécialisées (IRIS), APHP Sorbonne Université Hopital Tenon, Paris, France; 2 GRC Endometriose, Sorbonne Université, Paris, France; 3 Department of Imaging and Radiation Oncology, Fondazione Policlinico Universitario Agostino Gemelli IRCCS, Rome, Italy; 4 Chamie Imagem da Mulher, Sao Paulo, Brazil; 5 Imaging Department of Hospital da Luz Lisboa, Lisboa, Portugal; 6 Department of Imaging, Imperial College Healthcare NHS Trust, London, UK; 7 Department of Surgery & Cancer, Imperial College London, London, UK; 8 Cambridge University Hospitals, Cambridge, UK; 9 Department of radiology, Hospices Civils de Lyon, Lyon Sud University Hospital, Lyon 1 Claude Bernard University, EMR 3738, Pierre Bénite, France; 10 Department of Radiological, Pathological and Oncological Sciences, Sapienza University of Rome, Rome, Italy; 11 Department of Radiology, Instituto Portugues de Oncologia de Lisboa Francisco Gentil, Lisboa, Portugal; 12 Department of Diagnostic Radiology, IRCCS Foundation San Gerardo dei Tintori, Monza, Italy; 13 Department of Radiology, PMU, University Hospital of Salzburg, Salzburg, Austria; 14 Imaging Institute of Southern Switzerland (IIMSI), Ente Ospedaliero Cantonale (EOC), Lugano, Switzerland; 15 Faculty of Biomedical Sciences, Università della Svizzera Italiana (USI), Lugano, Switzerland; 16 Department of Radiology, Izmir City Hospital, University of Health Sciences, Izmir, Turkey; 17 Department of Radiology, LMU University Hospital, LMU Munich, München, Germany; 18 Hospital Clínic, Servei de Radiodiagnòstic, Barcelona, Spain; 19 Research Center for Medical Image Analysis and AI (MIAAI), Danube Private University, Krems, Austria

**Keywords:** Endometriosis (Pelvis), Magnetic resonance imaging, Consensus

## Abstract

**Objective:**

To propose an update of ESUR endometriosis guidelines to reflect advances in MRI protocol and lexicon.

**Methods:**

A literature search was followed by a DELPHI process among 20 experts.

**Results:**

Pre-imaging preparation, including fasting, antiperistaltic agents, moderate bladder filling, and bowel preparation, is recommended. A comprehensive magnetic resonance imaging (MRI) protocol should include multiplanar T2W, T1W, and sequences covering the kidneys. Superficial endometriosis should be described on T1WFS as high signal intensity foci on the peritoneal surface. Endometriomas should be described in terms of multiplicity, signal intensity, central or peripheral location, and bilaterality. MRI evaluation of deep pelvic endometriosis (DE) should be performed by dividing the pelvis into compartments using two horizontal and vertical lines. A bladder nodule should be described according to location, size, and the distance to the ureteric orifice provided. A uterosacral ligament must be considered abnormal if a nodule or spiculation is visible in at least two planes or if a bright T1W spot is detected. A posterior vaginal wall nodule should be measured. External adenomyosis should be described according to location and size. The description of a rectosigmoid nodule includes location, number of nodules, longitudinal extent, distance to the anal verge, and wall thickening. The lateral compartment includes the anterior distal round ligament, the mediolateral and posterolateral parametrium. Abdominal wall nodules, ileocaecal junction, appendiceal nodules, and sigmoid nodules, must be systematically described.

**Conclusion:**

A standardized MRI protocol and lexicon based on compartmental analysis are crucial for improving communication and management of patients referred with endometriosis.

**Key Points:**

***Question*** *ESUR’s endometriosis guidelines were last published in 2017; an update is provided to reflect advances in MRI techniques and the need for a standardized lexicon*.

***Findings*** *MRI protocol must include multiplanar T2W sequences, a T1W sequence, and a kidney visualization sequence. A standardized report based on a compartmental analysis is recommended*.

***Clinical relevance*** *Using a standard MRI protocol with compartmental analysis of endometriotic nodule locations and adopting a standardized vocabulary is crucial for comprehensive mapping and effective communication with both the patient and the surgeon*.

**Graphical Abstract:**

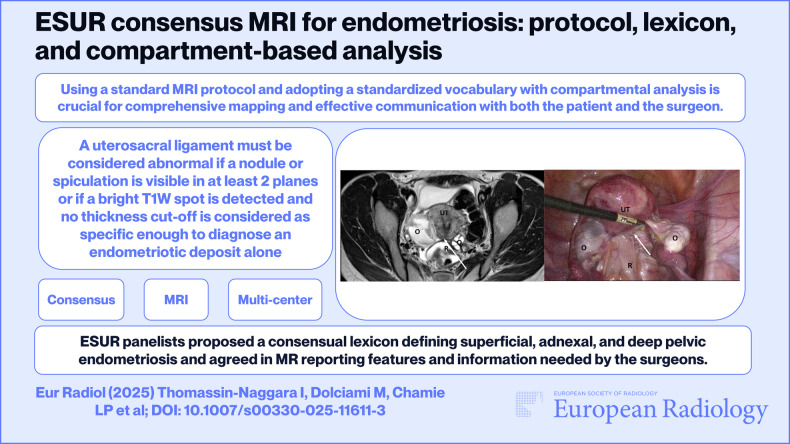

## Introduction

Endometriosis, defined by the presence of endometrium-like tissue outside the uterus, affects 2–10% of the female population, representing at least 190 million women worldwide [1, 2]. In addition, endometriosis is reported in 50% of women of reproductive age with chronic pelvic pain and or with a history of infertility [3], but this includes predominantly superficial endometriosis, which is often overlooked or not seen on imaging [4]. In contrast, adnexal endometriosis and deep pelvic endometriosis (DE) have a more aggressive behavior in some women with endometriosis.

In 2017, the first European Society of Urogenital Radiology (ESUR) consensus proposed some recommendations regarding patient preparation and imaging acquisition to start standardizing practice [5]. However, the MRI technique has improved with high-resolution 3D sequences and AI denoising solutions, necessitating a review of protocol guidelines [6].

Since the previous consensus [7–10], several authors have proposed a consensual lexicon and developed different reporting classifications to improve communication between the radiologist and the patient or the radiologist and the surgeon [11–15]. This is why it is time to change imaging guidelines in endometriosis [16].

## Methods

Delphi process and determining consensus are detailed in Supplemental Material 1

### Expert panel members

The panel included 20 experts from 10 different countries, including 14 European centers and one non-European institution (Italy (*n* = 5), France (*n* = 4), Austria (*n* = 2), Portugal (*n* = 2), United Kingdom (*n* = 2), Germany (*n* = 1), Spain (*n* = 1), Turkey (*n* = 1), Switzerland (*n* = 1) and Brazil (*n* = 1). Ninety percent of panelists (18/20) were affiliated with referral centers for endometriosis with a high volume of dedicated transvaginal ultrasonography (80% (16/20). In half of these centers, the radiologists themselves conduct the ultrasound examinations. Regarding MRI practice, more than 30 exams are acquired monthly in 50% of centers. Additionally, 50% of panelists (10/20) regularly participate in multidisciplinary meetings dedicated to endometriosis.

## Protocol

ESUR panelists confirm previous recommendations regarding MRI protocol published in the ESUR consensus in 2018. Technical advances have made it possible to obtain thinner slices without a gap and maintaining a short acquisition time, thus often eliminating the need for a third plane (which is useful in the case of partial volume) suggesting the usefulness of 3DT2 sequences with multi-planar reconstructions, even if the consensus has not yet reached 80% (Table 1).

**Table 1:** MR protocol

PREPARATION	Grade	Consensus agreement
MR scheduling for endometriosis: No specific timing in the menstrual cycle is recommended	C	95% (19/20)
Pre-imaging fasting is recommended	D	80% (16/20)
The use of anti-peristaltic agents* is recommended	D	95% (19/20)
A moderately filled bladder** is highly recommended	D	85% (17/20)
Bowel preparation*** before MR examination is highly recommended, either a bowel enema or a rectal suppository	B	80% (16/20)
Vaginal opacification (10cc) should be considered as an option and no systematically performed	B	75% (15/20)
Rectal opacification could be considered as an option, knowing that this may obscure adjacent lesions	B	85%, 17/20
There is no recommendation regarding the use of a specific magnet strength, phased array coils are highly recommended at 1.5 T and 3 T	C	(95%, 19/20)
Women should be placed in the supine position	C	100% (20/20)
SEQUENCES		
Fundamental		
Two different 2DT2W planes (sagittal and axial)	A	100% (20/20)
T1W/T1W-FS or 3D Dixon technique	A	100% (20/20)
A sequence that visualizes the Kidneys	A	95% (19/20)
Optional		
Thin T2W oblique slices or 3DT2W with reformat in USL plan	C	75%, 15/20
Diffusion-weighted imaging (adnexal mass characterization)	A	80%, 18/20
T1W with gadolinium injection (atypical endometriomas, abdominal wall endometriosis, nerve locations)	A	95%, 19/20
Upper abdomen up to the diaphragm	C	

^*^ Regarding the duration of efficacy of these drugs, using just before the most informative sequence (2DT2WI, 3DT2WI) is recommended

^**^ By voiding the bladder 30–60 min before the examination, following a drink of water

^***^ Bowel preparation options include a low-residue diet for three days, oral laxative the day prior, senna extract for three days, four doses of polyethylene glycol powder the prior night, or a suppository, rectal enema, or bowel evacuation just prior to the exam

## Lexicon and compartment-based analysis

In this section, we classify the different types of endometriosis, beginning with superficial endometriosis that corresponds to peritoneal implants, followed by adnexal endometriosis that describes any ovarian or tubal locations, and ending with DE which is defined as an extension of endometrial-like tissue under the peritoneal surface, usually nodular and able to invade adjacent structures, associated with fibrosis and disruption of normal anatomy, according to recent international consensus [17].

### Superficial endometriosis

ESUR panelists strongly agreed that superficial endometriosis should be described when high signal intensity foci on T1-weighted (T1W) with fat suppression (FS) are seen on the peritoneal surface without DE (100%, 20/20). In contrast, a consensus was not found among the experts to formally define superficial endometriosis in the presence of a peritoneal cystic lesion (60%, 12/20) or adhesion, defined as hypointense bands in T2-weighted (T2W) signal intensity that tether structures together (50%, 10/20).

All the experts agreed that the site of superficial endometriosis should be mentioned (100%, 20/20), including ovarian fossa, mesosalpinx, pouch of Douglas, and parietal peritoneum. However, only 50% of experts (10/20) provide a measurement in their report.

**Statement 1:** Superficial endometriosis should be described when bright spots on T1-weighted with fat saturation (T1WFS) are present on the peritoneal surface, and the location mentioned (Strong agreement).

### Adnexal endometriosis

#### **Definition**

Adnexal endometriosis may correspond to three entities: Micro-endometrioma, endometrioma, or endometriotic hematosalpinx. All these lesions demonstrate higher than fatty tissue on non fat sat T1W sequence. In endometriomas, a low T2W signal intensity is typically described as a “shading” sign [5].

#### **MRI key features**

The term “endometrioma” should be used for a cyst > 1 cm (ESUR expert group 85%; 17/20). The term micro-endometrioma is used for intraovarian high T1W FS signal intensity < or = 1 cm. The following descriptors should be used to describe an endometrioma or a micro-endometrioma: multiplicity (100; 20/20), signal intensity (95%, 19/20), central or peripheral location (90%, 18/20), and bilaterality (90%, 18/20).

**Fig. 1:**
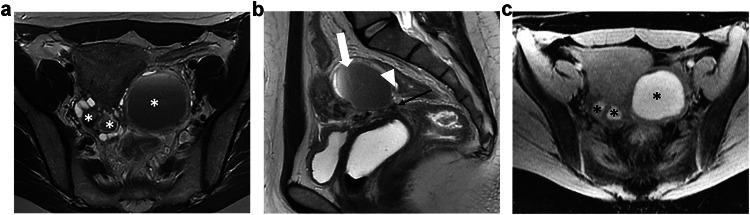
Bilateral ovarian endometriomas and kissing ovaries in a 35-year-old woman with severe pelvic pain. **a** Axial T2W MR image shows shading within the bilateral endometriomas (*). The ovaries are medially displaced in the retrouterine region. **b** Sagittal T2W MR image shows shading and fluid-fluid level within the left ovarian endometrioma (white arrow). There is also a low signal intensity thickening of the peritoneum of the ovarian fossa (black arrow) and tethering of the bowel serosa to the ovarian capsule (arrowhead). **c** Axial T1WFS MR image shows bilateral high-signal intensity cysts, likely due to hemorrhagic content (*)

An endometrioma appears as an unilocular cyst and is rated ovarian-adnexal reporting and data system (O-RADS) MR 2 due to endometriotic content (Low T2W, High T1W signal). If a solid component is depicted on morphological images, a gadolinium injection should be discussed, as emphasized by ESUR experts (Fig. 1a–c). Clots may mimic solid tissue on T2W sequences. If the clots are difficult to diagnose, the absence of enhancement after gadolinium injection enables accurate characterization. ESUR panelists agreed that the absence/presence of solid tissue must be reported in any description of an endometrioma (95%, 19/20). A typical endometrioma does not show any wall or internal enhancement, which allows the exclusion of any solid tissue. If solid tissue is suspected, a complete protocol for adnexal characterization should be performed, including diffusion-weighted and DCE MR sequences as recommended by the O-RADS MR scoring system [18]. Solid tissue present in an endometrioma may correspond to a malignant degeneration (seromucinous borderline transformation, clear cell tumor, or endometrioid cell tumor) that may occur in 1–2% of endometrioma [19].

Finally, a hematosalpinx or hydrosalpinx is suspected when dilated, fluid-filled tubular structures are depicted in the adnexa. Hydrosalpinx is described when the liquid content is high T2W signal and low T1W signal (serous), while endometriotic hematosalpinx is suspected when the content is high T1W signal equal to or superior to fat tissue and intermediate or low T2W signal. ESUR panelists stated that tubal content (serous, hemorrhagic, purulent), dimension (caliber), and wall thickening should be reported (90%, 18/20). Tubal folds must not be confused with solid tissue.

#### **Information needed by the gynecologist**

Regarding pain and fertility issues, ESUR experts confirm the necessity to measure any endometrioma with at least its maximal diameter (19/20) and to report the number of lesions (90%, 19/20), and the uni/bilaterality (90%, 19/20), as well as the presence of any dilated tube (including hydro and hematosalpinx) (90%, 18/20). The presence of residual ovarian parenchyma (with size and number of follicles) was also considered an important criterion to describe by a majority of experts, without reaching a consensus (70%, 14/20).

**Statement 2: adnexal endometriosis**

- The term micro-endometrioma may be used for intraovarian high T1W FS signal < or = 1 cm, and the report should include multiplicity, signal intensity, central or peripheral location, and uni/bilaterality (strong agreement).
- The term endometrioma should be used for a cyst > 1 cm with endometriotic content and without solid tissue. The report should include maximal diameter, multiplicity, central or peripheral location, uni/bilaterality, and any associated dilated tube (Strong agreement). The description of residual ovarian parenchyma (size and follicles) should be described (agreement).
- A hematosalpinx or hydrosalpinx is suspected when dilated fluid-filled tubular structures are seen in the adnexa; the tubal content and any wall thickening must be reported (strong agreement).

### Internal adenomyosis

Adenomyosis is a condition related to endometriosis, characterized by the presence of endometrial-like cells within the myometrium, which can lead to pelvic pain and infertility [2]. Internal adenomyosis should not be confused with external adenomyosis, in which endometriosis infiltrates the myometrium from the outside (described subsequently, in the DE paragraph). MRI diagnosis is based on direct signs (endometrial cysts within myometrium) and indirect signs (junctional thickening > 11 mm, asymmetry of anterior/posterior myometrium, ratio of JZ to overall myometrial thickness > 40%), and different classifications exist [20–22]. All ESUR experts agreed that the presence of internal adenomyosis should be described (100%, 20/20), describing the type (focal or diffuse) (95%, 19/20), or the presence of adenomyoma (85%, 17/20). The majority of ESUR experts also proposed to describe the symmetric/asymmetric subtypes (75%, 15/20), the superficial/deep adenomyosis (involvement of the junctional zone only, or involvement of the outer myometrium, respectively) (70%, 14/20), and the active/inactive disease (presence of microcyst in highT2W and/or highT1W with fat saturation) (70%, 14/20) without reaching a consensus.

**Statement 3:** Internal adenomyosis is an associated disease that should be described in an MRI report for endometriosis (strong agreement), describing the type (focal, diffuse adenomyoma) (strong agreement), symmetric/asymmetric subtypes, superficial/deep adenomyosis, and active/inactive disease (simple agreement).

### Deep pelvic endometriosis

#### General recommendations

ESUR panelists strongly agreed with the necessity to describe the different possible imaging findings that may constitute DE: glandular or cystic component (95%, 19/20), fibrosis (90%, 18/20), and adhesions (90%, 18/20). In addition, the majority of the ESUR expert group suggested describing the presence/absence of microcysts with high T2W signal and high T1W signal within the disease (75%, 15/20).

Finally, they strongly stated that when a radiologist evaluates an MRI for endometriosis, the pelvis should be divided into compartments (95%, 19/20) as previously suggested by different international and national societies [9, 14]. The ESUR expert group agreed on the following compartments: anterior, posterior, medial, lateral, and extra-pelvic (95%, 19/20).

**Fig. 2:**
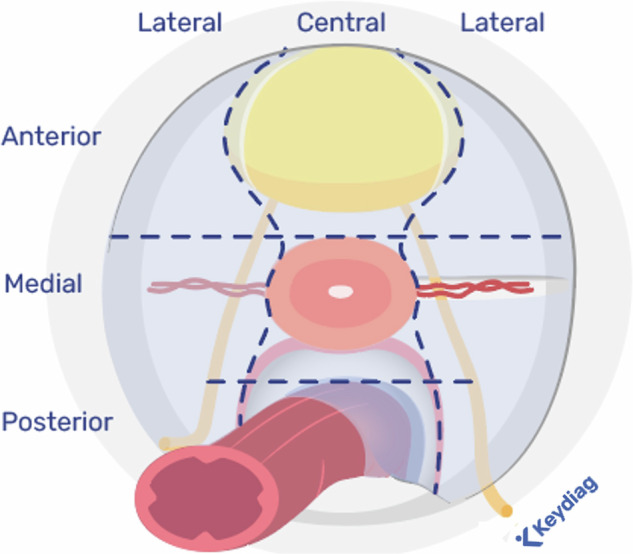
Compartmental analysis
- Extra-pelvic locations correspond for ESUR panelists to the abdominal wall (100%, 20/20), sigmoid colon (85%, 17/20), ileocecal junction/appendix (80%, 16/20), and the inguinal canal (75%, 15/20). The diaphragm is also an extra-pelvic location but not systematically included in a classical protocol by many experts from the ESUR group (60%, 12/20).

- Anterior, medial, and posterior compartments are defined by two figurative straight horizontal lines, with an anterior line passing anterior to the cervix or vagina and a posterior line passing anterior to the rectum [14].
- Lateral compartments are medially delineated by two figurative curved ventrodorsal lines, each line passing from posterior to anterior along by the uterosacral ligament (USL) and the underlying mesorectal fascia, the lateral border of the uterine cervix or vagina, and the lateral wall of the bladder [14] (Fig. 2).

Although the compartments do not correspond to defined anatomical spaces, their identification is important for report standardization and enhancing communication with clinicians and patients [11, 14, 16].

**Statement 4:** MRI evaluation of DE should be performed using a compartmental division (strongly agreement) defining two horizontal lines with an anterior line passing anterior to the cervix or vagina and a posterior line passing anterior to the rectum and two vertical lines passing from back to front by the USL and the underlying mesorectal fascia, the lateral border of the uterine cervix or vagina, and the lateral wall of the bladder.

Table 2 extensively describes the typical sites of pelvic DE according to the compartmental division and how to recognize their involvement.

**Table 2:** MR diagnostic criteria for DE sites

CENTRAL LOCATIONS					
A N T E R O C E N T R A L	Bladder: suspected • When a nodule/mass forms an obtuse angle with the bladder wall involving the muscular layer (obliteration of the hypointense wall signal in T2W). • Protrudes into the lumen, invading the mucosal layer. • Located on dome (1), level of CVU (2), or basis (3).				
	Proximal round ligament: suspected • When the ligament shows fibrotic thickening compared to the contralateral round ligament, with regular or irregular margins and occasionally a nodular appearance. • The proximal tract arises from each uterine horn and corresponds to the intrapelvic one-third of the round ligament.				
M E D I O C E N T R A L	Torus/proximal USL: suspected • In the presence of a mass or thickening in the upper/mid-portion of the posterior cervix. • If there is a unilateral USL nodule or fibrotic thickening compared to the contralateral USL, with regular or irregular margins. • An “arciform abnormality” is described when the involvement of the torus uterinus affects both USLs.				
	Retrouterine space or pouch of Douglas (RUP): suspected when there is a partial or complete obliteration of the retrouterine space, with/without a suspended or lateralized fluid collection.				
	External adenomyosis (EA): reported in the presence of a nodular hypointense extrinsic infiltration of the outer myometrium on T2W images with ill-defined borders along the anterior/posterior uterine wall.				
	Vagina: suspected if there is partial replacement of the posterior vaginal wall/posterior vaginal fornix on T2W images, with a hypointense thickening or a mass (with or without foci of high T2W SI) behind the infero-posterior wall of the cervix.				
	Rectovaginal space: suspected when a nodule or mass passes the lower edge of the peritoneal reflection (typically located at the level of the posterior lip of the cervix).				
P O S T E R O C E N T R A L	Rectum/rectosigmoid junction (RS jct): suspected • When there is focal hypointense thickening or mass of the anterior wall of the rectum/sigmoid colon on T2W images. • Located on high, middle, and/or low rectum or at the level of the rectosigmoid junction.				
LATERAL LOCATIONS					
A N T E R O L A T E R A L	Distal round ligament: • Suspected when the ligament shows fibrotic thickening compared to the contralateral round ligament, with regular or irregular margins and occasionally a nodular appearance. • The distal round ligament corresponds to the intrapelvic distal two-thirds of the round ligament, up to the deep orifice and/or into the inguinal canal.				
M E D I O L A T E R A L	Mediolateral parametrium: suspected • When there is irregular and/or retractile fibrotic infiltration, with or without hemorrhagic cystic areas, within the vascular and adipose tissue of the lateral or posterolateral cervical or vaginal regions. • This typically arises from a nodular lesion (over 1 cm) in the proximal USL extending downwards and outwards or from a lesion in the inferior and posterior broad ligament.				
	Ureter: suspected • When there is fibrotic infiltration of the ureteral wall and often peri-ureteral fat (focal low signal intensity), typically where the ureter enters the parametrium or, more posteriorly, along the broad ligament’s posterior leaf.				
	Nerves involved: Obturator nerve (L2–L4).				
P O S T E R O L A T E R A L	Posterolateral parametrium: suspected • When there is a fibrotic nodular infiltration within the lateral pelvic compartment, behind the posterior horizontal line passing anteriorly to the rectum, either unilaterally or bilaterally, with loss of the normal appearance of the retro and subperitoneal pararectal space located outside the mesorectal fascia. • The USL represents the roof of the sacrorectal septum, therefore, an endometriotic deposit that infiltrates underneath and outside the USL into the pararectal space invades the sacrorectal septum.				
	Nerves involved: lumbosacral plexus and/or roots (S2, S3, and S4), sciatic nerve (L4–S3)				
EXTRA PELVIC LOCATIONS					
WALL		Abdominal-pelvic wall nodules			
DIGESTIVE		Sigmoid nodules Ileocecal junction Appendiceal nodules			
DIAPHRAGM		Diaphragmatic deposits			

#### Central locations

**Fig. 3:**
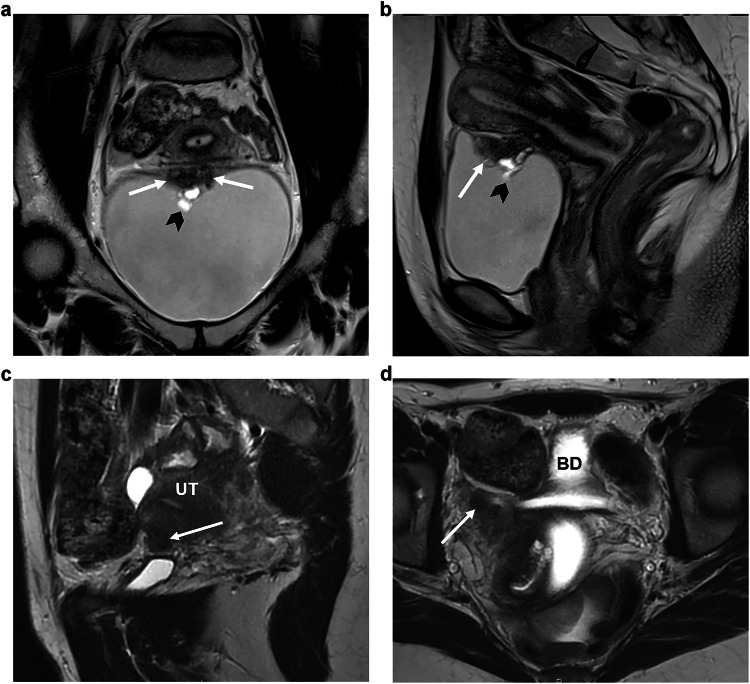
Bladder and round ligament endometriosis. **a**, **b** Bladder endometriosis in a 29-year-old woman with dysuria. The coronal (**a**) and sagittal (**b**) T2W MR images reveal a well-defined, low-signal-intensity nodule (arrows) in the bladder wall projecting into the lumen. The nodule exhibits heterogeneous content due to small cystic areas indicative of glandular components (arrowhead). **c**, **d** Endometriosis affecting the proximal third of the right round ligament in a 27-year-old woman with chronic pelvic pain. The sagittal (**c**) and axial (**a**) T2W MR images demonstrate low-signal intensity nodular thickening of the round ligament at its uterine insertion site (arrows). UT, uterus; BD, bladder

***Anterior compartment:*** **Bladder**: *MRI reporting features*: when there is bladder involvement, ESUR experts strongly agreed that the following three bladder wall locations should be described: the anterior two-thirds of the bladder dome, the posterior third of the bladder dome at the level of the vesico-uterine space (VUS) and the base which includes the trigone between the ureteral orifices (95%, 19/20, and 80%, 16/20, respectively) (Fig. 3a–d).

*Information needed by the surgeons*: ESUR panelists strongly agreed that the nodule should be measured according to an anteroposterior diameter (95%, 19/20); only approximately half of the panelists suggested that the laterolateral (60%, 12/20) and craniocaudal dimensions (65%, 13/20) should also be measured. In addition to the size and the description of the lateralization of the lesion (midline, right-sided, or left-sided), the majority of the ESUR expert group indicated that providing the distance to the ureteral orifice is valuable information to give within the MRI report (75%, 15/20)

**Statement 5:** A bladder nodule has to be localized and measured anteroposteriorly, and VUS involvement has to be specified (Strong agreement). The distance to the ureteral orifice should be provided (simple agreement).

**Proximal round ligament**: *MRI reporting features*: ESUR panelists stated that the proximal round ligament should be measured along the transverse axis (16/20, 80%), identified by the latero-lateral diameter in an axial or coronal plane. However, no agreement was found regarding a strong MRI criterion to accurately diagnose an endometriotic deposit on proximal round ligament: when the thickness is greater than 10 mm in proximal one third (9/20), only if involved by a nodule of DE (8/20), always when thickened (3/20), or only if there are bright spots in T1WFS (5/20)

***Medial compartment:*** the authors use various terms to refer to this compartment, including “rectovaginal/rectouterine” or “retrocervical” and “space” or “area.” This compartment is the most frequently involved by endometriotic deposits.

**Fig. 4:**
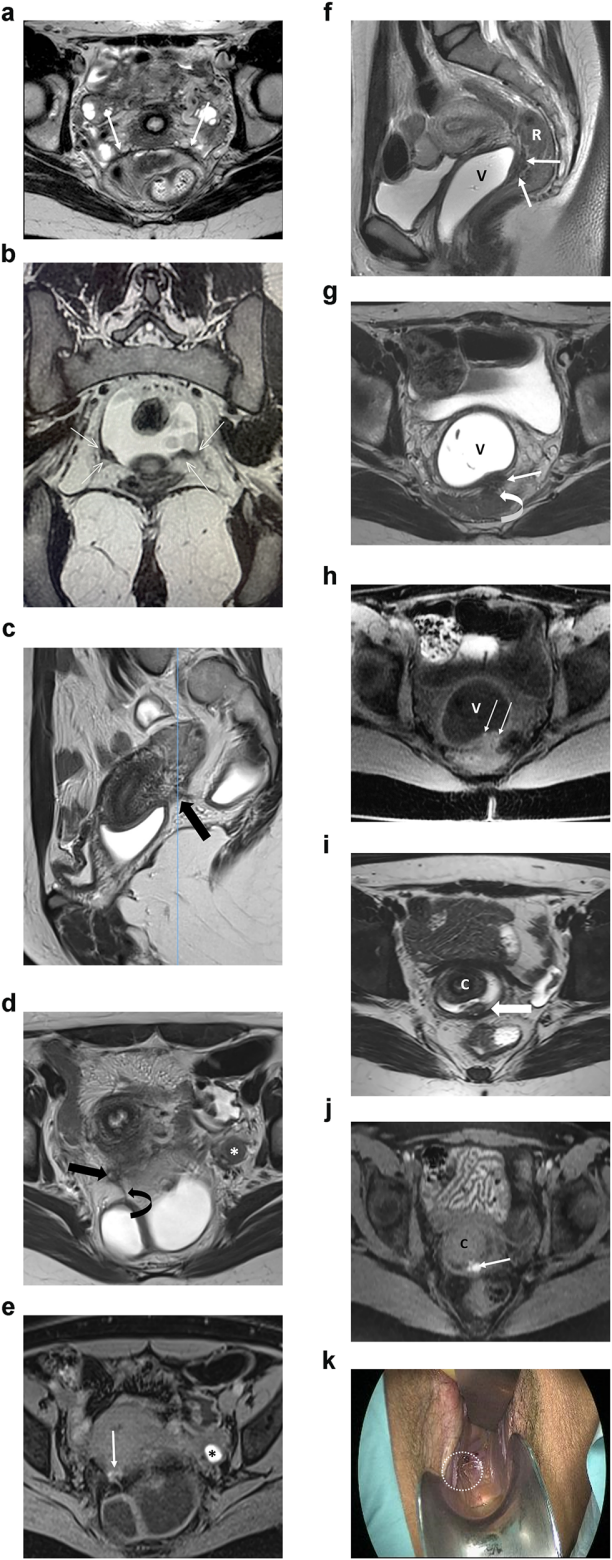
Different imaging examples of endometriosis affecting the torus uterinus and USLs. **a** Bilateral involvement of the USLs in a 35-year-old woman with dyspareunia and pelvic pain. The axial T2W MR image shows proximal USL thickening with low signal intensity and irregular margins (white arrows), known as an arciform pattern. **b** Bilateral involvement of the USLs in a 17-year-old woman with severe dysmenorrhea. The coronal T2W image demonstrates asymmetric thickening of the proximal thirds of the USLs (arrows). **c**–**e** Endometriosis affecting the right USL in a 27-year-old woman with dysmenorrhea and dyspareunia. The sagittal (**c**) and axial (**d**) T2W MR images show nodular thickening of the proximal third of the right USL (arrows). There is also a fibrous septum attached to the adjacent bowel serosa (curved arrow in d) and a small left ovarian endometrioma (* in **d**). The axial T1WFS MR image (**e**) depicts hyperintensity foci within both the uterosacral thickening (arrow) and the ovarian endometrioma (*). **f**–**h** Endometriosis affecting the rectovaginal septum in a 35-year-old woman with dyspareunia. The sagittal (**f**) and axial (**g**) T2W MR images demonstrate nodular thickening with low signal intensity of the rectovaginal septum (arrows), associated with adhesion to the anterior rectal wall (curved arrow in **g**). The axial T1WFS MR image (**h**) shows tiny hyperintensity foci within the nodule (arrows). **i**–**k** Vaginal endometriosis in a 30-year-old woman with deep dyspareunia. The axial T2W MR image (**i**) shows nodular thickening occupying the posterior vaginal fornix (arrow). The axial T1WFS MR image (**j**) depicts hyperintensity foci within the nodule (arrow). Image (**k**) obtained during vaginal examination demonstrates endometriotic infiltration of the posterior vaginal fornix (white circle). R, rectum; V, vagina; C, cervix

**Torus uterinus and proximal USL**: The torus uterinus corresponds to the fusion of the USLs posteriorly to the cervix that forms a semicircle denoting the borders of the posterior cul-de-sac (pouch of Douglas). Thus, the proximal USL is located along the posterior vaginal fornix and corresponds to the segment with a transverse orientation (Fig. 4a–k). The conventional elements defining the MRI location of the torus/USL ligament were deemed insufficiently precise, and many publications demonstrated their lack of reproducibility [23]. Thus, the group went further in the description of MRI criteria to positively identify a torus/USL deposit (Table 2).

*MRI reporting features*: ESUR panelists agreed that a USL is normal if its thickness is ≤ 3 mm (80%, 16/20). Most of the ESUR experts reported that they measured the transverse thickness (85%, 17/20), but few of them considered it useful to measure the length of a thickness (5%, 4/20). They also strongly agreed that a USL must be considered abnormal if a spiculation or nodule is visible at least in two planes at least (100%, 20/20) or if a T1W bright spot is detected (80%, 16/20). Finally, ESUR panelists considered that a deposit of endometriosis should be considered as uncertain if a spiculation or nodule is only visible in one plane (90%, 18/20), and no thickness cut-off was considered specific enough to diagnose an endometriotic deposit alone.

*Information needed by the surgeons:* Most of the ESUR panelists agreed that the description of the site (proximal or distal) of the deposit should be detailed (70%, 14/20). The infiltration of the mesorectum is also an important element to report, especially if the infiltration extends laterally, as this makes the surgery more complex.

**Statement 6:** A USL is considered normal if its thickness is ≤ 3 mm. A USL must be considered abnormal if a spiculation or nodule is visible in at least two planes or if a T1W bright spot is detected. A USL must be considered as uncertain if a spiculation or nodule is only visible in one plane (strong agreement).

**Posterior vaginal wall**: *MRI reporting features*: ESUR panelists strongly agreed that an involvement of posterior vaginal wall must be mentioned (100%, 20/20).

*Information needed by the surgeons:* ESUR panelists also strongly agreed that an anteroposterior and/or craniocaudal measurement should be given (80%, 16/20)

**Rectouterine space or pouch of Douglas**: *MRI reporting features*: ESUR panelists agreed on the requirement to report the involvement of the pouch of Douglas (100%, 20/20).

*Information needed by the surgeons:* ESUR panelists also strongly agreed that an anteroposterior and/or craniocaudal measurement should be given (85%, 17/20).

**Rectovaginal septum**: the rectovaginal space is a retroperitoneal location corresponding to the space between the vagina and the anterior wall of the mid and lower rectum below the peritoneal reflection of the pouch of Douglas.

*Definition and MRI reporting features*: the ESUR expert group strongly agreed that this term “rectovaginal septum deposit” must be solely used for sub-peritoneal or retroperitoneal (below the pouch of Douglas peritoneal reflection) nodules (85%, 17/20).

*Information needed by the surgeons:* length, thickness, and distance to the anal verge should be mentioned.

**Uterus: external adenomyosis or myometrial infiltration**: external adenomyosis is defined as the presence of endometrial glands and stroma with muscular reactive hyperplasia deeply infiltrating into the external myometrium with an outside-in pattern [5].

*MRI reporting features*: ESUR panelists strongly agreed that the presence of external adenomyosis should be reported (90%, 18/20)

*Information needed by the surgeons*: most of the ESUR experts recommended describing the depth of myometrial invasion: moderate (limited to the subserosal external myometrium) or severe (up to the junctional zone or more) (70%, 14/20), but only 25% (5/20) report the distance from the endometrium.

**Statement 7:** A posterior vaginal wall nodule or pouch of Douglas nodule should be measured according to anteroposterior and/or craniocaudal diameter. The term rectovaginal septum must be utilized only for retroperitoneal nodules (strong agreement). External adenomyosis should be described (strong agreement) and measured (simple agreement).

**Fig. 5:**
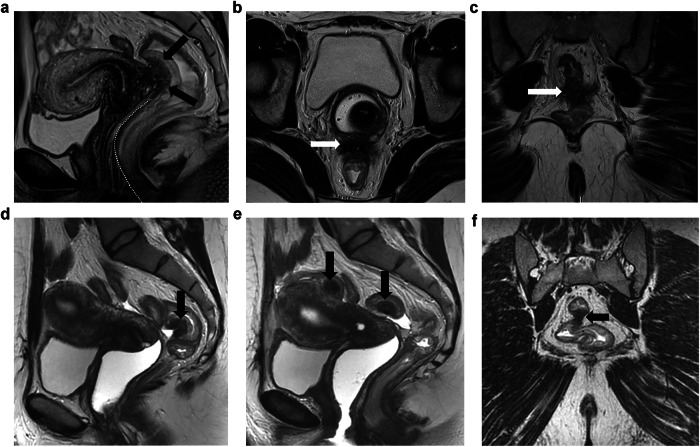
Rectal and sigmoid endometriosis. The figure illustrates the presence of a mass with low signal intensity affecting the anterior wall of the rectum with a typical “mushroom cap” pattern on sagittal T2W MR image (**a**), the low intensity base surrounded by a cap of high signal intensity on T2W sequence represented the edema of the sub-mucosa/mucosa displaced to the bowel lumen (arrow). The spiculated endometriotic nodule also infiltrates the rectovaginal septum and posterior vaginal wall illustrated on axial (**b**) and coronal (**c**) T2W MR images (arrows). The distance between inferior margin of the rectal nodule and anal verge must be measured as represented by the dashed line on sagittal T2W MR image (&^HJYU**a**). The sagittal T2W MR images (**d**, **e**) and coronal T2W MR image (**f**), represent another patient with multiple endometriosis rectal and rectosigmoid low signal wall nodules (arrows)

***Posterior compartment:*** **Rectum/rectosigmoid junction**: the location of the rectal lesion is reported according to the distance between the anal verge and the mid-point of the rectal lesion, as follows: upper rectum (> 10 to 15 cm), mid rectum (> 5 to 10 cm), and lower rectum (0 to > 5 cm). Lesions located 15 cm above the anal verge or at the site of the sigmoid take-off are attributed to the recto-sigmoid junction (Fig. 5a–e).

*MRI reporting features*: ESUR panelists strongly agreed to report the rectal portion involved (lower, middle, upper) (90%, 18/20) and the number of nodules (80%, 16/20).

*Information needed by the surgeons*: ESUR 3epanelists strongly agreed to report longitudinal extent (90%, 18/20), distance to the anal verge (85%, 17/20), and depth of wall thickening (85%, 17/20). Most of the ESUR experts also reported the necessity to describe the transverse axis or involved circumference (70%, 14/20), while a minority of them reported submucosal or mucosal edema (40%, 8/20).

**Statement 8:** The description of a rectal/rectosigmoid nodule needs to include: location (lower, middle, upper rectum), number of nodules, longitudinal extent, distance to the anal verge, and wall thickening (strong agreement) and transverse axis or circumference (simple agreement).

#### Lateral locations

Lateral compartments are located on the right and left sides of the pelvis and contain, anteriorly, the distal portion of the round ligament, medially, the mediolateral parametrium (including ureter and uterine artery), and posteriorly, the posterolateral parametrium (including distal USL, sacrorectal septum).

**Distal round ligament:** the round ligament crosses the pelvis through the deep inguinal ring, then traverses the inguinal canal and enters the labia majora and terminates into the mons pubis, following the canal of Nuck. Involvement at the superficial orifice of the inguinal canal is considered in the extra-pelvic compartment, being part of the perineum, and may require a perineal approach.

*MRI reporting features*: ESUR panelists stated that distal round ligament thickening should be measured transversely (16/20, 80%). However, no agreement was established regarding a strong MRI criterion for a positive diagnosis of a distal round ligament endometriotic deposit. Suggested features include: when the ligament thickness is greater than 10 mm in the proximal portion (9/20), only if involved by a focal nodule of DE (8/20), always when thickened (3/20), and only if there are bright spots in T1WFS (5/20).

*Information needed by the surgeon*: the nodule can be near the femoral nerve and external iliac vessels, and these elements must be reported.

**Medio-lateral parametrium**: the mediolateral parametrium corresponds to connective tissue forming a sheet containing blood vessels, the ureter, and the inferior hypogastric plexus (beneath the ureter and deep uterine vein). It extends from the lateral surface of the cervix and vagiuna to the lateral pelvic wall in the coronal plane. On MRI, the proximal parametrium is identified medially to the ureter, while the distal parametrium is identified laterally to the ureter out to the pelvic sidewall [16], offering an increased surgical risk [24].

*MRI reporting features*: ESUR panelists strongly agreed that medio-lateral parametrial invasion should be described (90%, 18/20).

*Information needed by the surgeons*: the description of parametrial involvement is crucial, as the risk of hypogastric plexus injuries is important, and the patient should be informed of the risk of de novo dysuria. Moreover, the extension to the uterine artery should also be evaluated to predict the risk of hemorrhagic complications.

**Ureter:** *MRI reporting features*: ESUR panelists strongly agreed that the diameter of the proximal ureter should be mentioned in any MRI report (80%, 16/20).

*Information needed by the surgeons*: ESUR panelists also strongly agreed that the distance from the ureteral endometriosis to the ureterovesical junction should be provided (80%, 16/20), a majority of them described the length of the involved ureter (70%, 14/20) but only a minority evaluated the involved circumference (> or < 180°) (35%, 7/20).

**Posterolateral parametrium: Distal USL/sacrorectal septum:** *MRI reporting features*: ESUR panelists strongly agreed that the description of postero-lateral parametrium should be reported (90%, 18/20). This space contains a large part of the hypogastric plexus, which, if injured during surgery, may result in many sympathetic dysfunctions, including de novo dysuria. The description of endometriosis at this site is therefore crucial.

*Information needed by the surgeons*: in addition, when a sacrorectal septum location is described, ESUR panelists strongly agreed that the distance or extension to the iliococcygeus muscle of levator ani was important to describe (80%, 16/20).

**Statement 9**: The lateral pelvic compartment can be further subdivided into anterior, medial, and posterior. Endometriosis within these locations comprises: involvement of the distal round ligament anterolaterally, the parametrium and ureter mediolaterally, and distal USL/sacrorectal ligament posterolaterally (strong agreement).

#### Nerve involvement

Endometriotic disease may damage somatic nerves and vessels. The five most frequently affected nerves are the lumbosacral plexus and/or roots (S2, S3, and S4), sciatic nerve (L4–S3), pudendal nerve (S2–S4), obturator nerve (L2–L4) (ventral division), and femoral nerve (L2–L4) (dorsal division) [25]. Muscles (including the levator ani, piriformis, and internal obturator muscles) and vessels may also be affected and therefore require evaluation.

## Extra pelvic locations

**Fig. 6:**
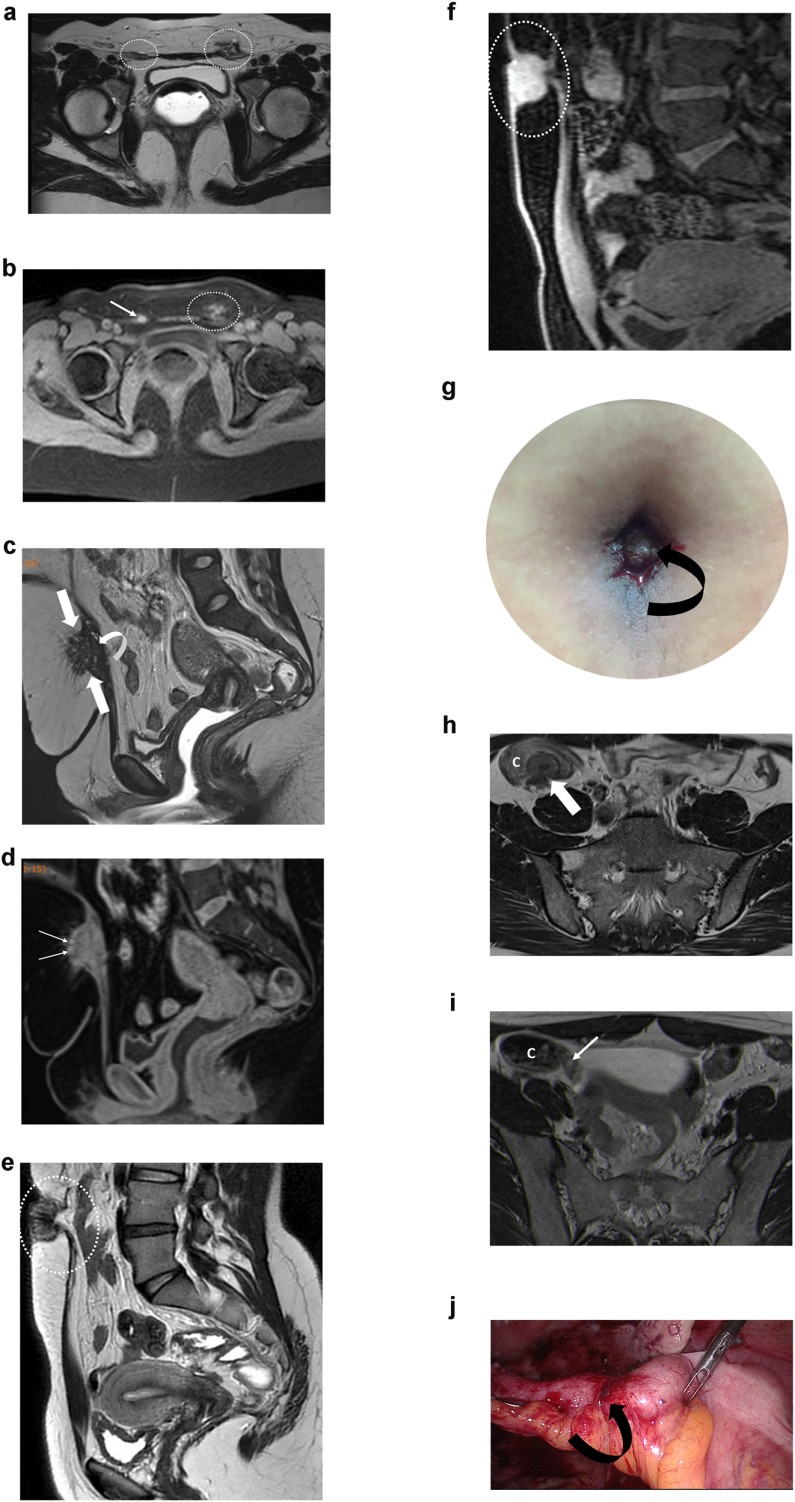
Different examples of extra-pelvic locations of endometriosis. **a**, **b** Bilateral inguinal endometriosis in a 31-year-old woman who presented with bilateral inguinal catamenial pain. **a** Axial T2W MR image shows nodular lesions (areas inside the circles) infiltrating the inguinal portions of the round ligaments. **b** Axial T1WFS MR image shows high signal intensity foci, likely due to hemorrhagic content (arrow and area inside the circle). **c**, **d** Cesarean scar endometriosis in a 40-year-old woman with pelvic pain. **c** Sagittal T2W MR image shows a nodule (arrows) with heterogeneous signal intensity, irregular margins, and internal areas of high signal intensity infiltrating the subcutaneous tissue and the rectus abdominis muscle (curved arrow). **d** Sagittal T1WFS MR image shows high signal intensity foci within the nodule, likely due to hemorrhagic content. **e**–**g** Umbilical endometriosis in a 31-year-old woman with cyclical bleeding and swelling in the umbilicus. **e** Sagittal T2W MR image shows a low signal intensity nodule infiltrating the umbilical scar (area inside the circle). **f** Sagittal T1WFS MR image shows a high signal-intensity umbilical nodule (area inside the oval) due to hemorrhagic content. Physical exam (**g**) shows an enlarged umbilical scar displaying a wine-colored appearance (curved arrow) associated with active bleeding. **h** Axial T2W MR image shows a low signal intensity nodule infiltrating the cecum (arrow) in a 32-year-old woman with multifocal pelvic endometriosis. **i** Axial T2W MR image shows a small nodule attached to the appendiceal base (arrow). Laparoscopic (**j**) resection confirmed an endometriotic implant (curved arrow). C, cecum

The ESUR panelists strongly agree that the following extra pelvic locations should be described in a pelvic MRI: abdominal wall nodules (100%, 20/20), ileocaecal/appendix (90%, 18/20), and sigmoid nodule (85%, 17/20). The ESUR panelists do not agree that evaluation of the diaphragm for endometriotic deposits should be performed systematically (60%; 12/20) (Fig. 6a–j).

**Statement 10:** Abdominal wall nodules, ileocaecal junction, appendiceal and sigmoid nodules have to be systematically described (strong agreement). The investigation of diaphragmatic endometriosis requires a dedicated sequence, and no agreement was obtained to recommend adding it routinely.

In conclusion, an MRI report should describe superficial endometriosis, adnexal endometriosis, and deep endometriosis according to a compartment-based analysis, facilitating a complete exhaustive mapping.

## Supplementary information

ELECTRONIC SUPPLEMENTARY MATERIAL

## Abbreviations

DE: Deep pelvic endometriosis
ESUR: European Society of Urogenital Radiology
MRI: Magnetic resonance imaging
O-RADS: Ovarian-adnexal reporting and data system
T1WFS: T1-weighted with fat saturation
T2W: T2-weighted
USL: Uterosacral ligament
