# Supplementary material for: ESUR consensus MRI for endometriosis: protocol, lexicon, and compartment-based analysis
Source: Eur Radiol. 2025 May 27;35(11):7272–86. doi: 10.1007/s00330-025-11611-3 (PMC12559084; doi:10.1007/s00330-025-11611-3)
Supplement: Supplementary file 1 — ELECTRONIC SUPPLEMENTARY MATERIAL [file 330_2025_11611_MOESM1_ESM.pdf]

ESUR Consensus MRI for Endometriosis: Protocol, lexicon, and compartment-based analysis.

ELECTRONIC SUPPLEMENTARY MATERIAL

Supplemental material 1: Delphi process and determining consensus

The ESUR Research Committee appointed two chairs (ITN, LM) to supervise the development of the updated guidelines. The updating process comprised the following steps:

Step 1—Literature Search: Four authors (ITN, LM, MD, and PR) performed a literature review of new data covering the time frame between 2017 and 2023 by searching PubMed/Medline database, EMBASE, and Cochrane Library for original articles in the English language on human subjects, including the following keywords: “endometriosis” AND “MRI” OR “Magnetic Resonance”. Appendix A shows the search strategy and the main results.

Step 2—Panel selection: The chairpersons selected 20 ESUR Female Pelvic Imaging Working Group (FPI-ESUR) members based on their competence in the field; the criteria for inclusion in the group were radiologists with at least five years of experience in endometriosis imaging and who had produced relevant indexed and peer-reviewed publications.

Step 3—Template development: The two chairs devised draft Delphi questionnaires, which the guideline committee further improved and approved. A final questionnaire was developed with 88 questions focusing on the following topics: characteristics of the examined imaging centers, indications for MRI examination, patient preparation, MRI protocol and technical details, analysis of MRI results, MRI lexicon, and reporting.

Step 4—Survey delivery: In November 2023, the questionnaire was distributed electronically to all panel members before the first meeting and responses were recorded.

Step 5 – Data extraction and analysis: The survey responses were collected in February 2024 and analyzed; each item was classified as follows: “RECOMMENDED” (if agreement ≥ 80%); “OPTIONAL” (if agreement ≥ 70 % but < 80 %); or “NOT RECOMMENDED” (if consensus was not reached, with < 70 % agreement). The results were presented and discussed during a virtual meeting in April 2024.

Step 6—Second survey: A new questionnaire version was emailed to all panel members to clarify any potentially conflicting answers that arose during the first survey round and the first meeting, and the new responses were recorded.

Step 7 – Second and final meeting: The panel members met again in June 2024 to discuss the remaining open questions on the various items. The focus was on the questions that created debate among the experts at the first meeting.

Step 8—Data reporting: The final survey responses were collected and analyzed in June 2024. Six authors (ITN, LM, MD, PR, AG, and LC) then prepared the first draft of the new recommendations, which also included the statements derived from the results obtained.

The first draft was shared with the group in August 2024, for possible suggestions or improvements. The final version of the recommendation paper was proposed in September 2024.

There is no accepted, set standard for the target percentage of agreement (26), and while 70% (summative of agree (≥70%) and strongly agree (≥80%)at the second round) is commonly reported in the literature, given the importance of promoting appropriate recommendations, the consensus was deemed to have been met at 80% (summative of agree and strongly agree) for each individual statement. If the percentage obtained at the second round was lower than 70%, the term disagree was considered.

The group were also asked to grade the level of evidence using the GRADE system on the same model as previous guidelines with the addition of publications between 2017 and 2024 (27).

Supplemental material 2: Protocol

Preparation

Preparation is crucial to improve image quality and will be detailed in this section including all elements that may impact MR diagnostic performance. MR examination is usually improved by abdominal strapping with an elastic belt (5).

Timing of MRI Examination

The previous ESUR consensus reported that discrepant results existed in the literature regarding the timing of MRI evaluation in relation to the menstrual cycle to improve detection, characterization, and assessment of disease extent (5). The ESUR expert group confirms that no specific scheduling of MRI in relation to the menstrual cycle for evaluating DE should be recommended (ESUR expert group, 95% (19/20).

Statement 2: Regarding MR scheduling for endometriosis, no specific timing in the menstrual cycle is recommended (Grade C, Strong agreement)

Fasting

Given that when fasting decreases, the peristalsis and thus digestive movement artifacts increase with an impact on image quality, most of the ESUR expert group (80%, 16/20) recommended a pre-examination fast with variable duration of 3, 4, and 6 hours, even if most studies did not specify any pre-imaging fasting.

Statement 3: Pre imaging fasting is recommended (Grade D, Strong agreement)

Anti-Peristaltic Agent

The use of an anti-peristaltic agent (e.g., glucagon, butyl-scopolamine), unless contraindicated (e.g., diabetes, pheochromocytoma, glaucoma, and mitral valve prolapse), is the most efficient way to limit uterine contractions and bowel motion artifacts (5,28). In line with the previous consensus (LE4), the ESUR expert group agreed regarding the usefulness (95% (19/20) of either intravenous or intramuscular administration. Half of the expert group (10/20) reported rare complications including tachycardia and more frequently visual impairments. Regarding the duration of efficacy of these drugs, using just before the most informative sequence (2DT2WI, 3DT2WI) is recommended.

Statement 4: The use of anti-peristaltic agents is recommended (Grade D, Strong agreement)

Bladder Emptying

The previous consensus underlines that a moderately filled bladder helps to detect small nodules located anterior to the vesicouterine pouch and displaces the bowel superiorly, reducing artifacts caused by bowel motion (LE4). In contrast, excessive bladder distension is not recommended as it may cause artifacts and complicate the identification of small parietal nodules (LE4). ESUR panelists highly agreed (85%, 17/20) that a recommendation for patients to void their bladder 30-60 minutes before the examination, following a drink of water, improves the detection of endometriotic nodules.

**Statement 5:** A moderately filled bladder is highly recommended (Grade D, Strong agreement)

Bowel Preparation

Bowel preparation options include a low residue diet for three days, oral laxative the day prior, senna extract for three days, four doses of poly-ethylene glycol powder the prior night, or a suppository, rectal enema, or bowel evacuation just prior to the exam. Previous European and American guidelines were discrepant regarding this recommendation due to the lack of evidence in the literature (5,28). Recently, a large multicentric study demonstrated that bowel preparation improves the diagnosis of endometriosis located on the uterosacral ligaments and rectosigmoid wall (29) (LE2). ESUR expert group highly recommended bowel preparation (80%, 16/20) even if no consensus was obtained regarding the method 12/16 experts recommended either bowel enema or a rectal suppository. Additionally, there was variability in dietary preparation with oral laxatives and a low-residue diet only recommended by 4 out of 16 experts.

**Statement 6:** Bowel preparation before MR examination is highly recommended (Grade B, strong agreement) either bowel enema or rectal suppository

Vaginal Opacification

To administer the vaginal contrast, a syringe is filled with 10 cc of aqueous gel and inadvertent air bubbles are expelled (28) . Previous ESUR consensus did not comment on the value of vaginal opacification (5) . A recent study underlines the absence of significant added value of vaginal opacification, especially compared with the use of 3DT2W and T1W sequences (LE3) (6). Furthermore, another study performed in approximately 550 women show that vaginal opacification could decrease the ability to correctly diagnose uterosacral endometriotic location, especially if the quantity of gelly is 20cc or more (LE2) (29). However, no consensus was reached regarding vaginal opacification with gel (15 out of 20 experts do not systematically administer vaginal contrast).

**Statement 7:** Vaginal opacification (10cc) should be considered as an option (Grade B, moderate agreement)

Rectal Opacification

Studies have demonstrated conflicting results as rectal distension with contrast may improve the detection of small nodules. Conversely, rectal contrast also increases motion artifact from peristalsis and may obscure lesions by compression against adjacent pelvic organs and neither study recommends it systematically (5,28). In line with American and European consensus, the ESUR expert highly agreed that rectal opacification should not be systematically performed (85%, 17/20). However, a recent study (29) demonstrated the value of rectal opacification if bowel preparation was not performed (LE2)

**Statement 8:** Rectal opacification could be considered as an option knowing that this may obscure adjacent lesions (Grade B, strong agreement)

MR protocol

Magnet strength, array coil, patient position

In 2018, the ESUR consensus reported only a few publications using 3.0T but suggested promising results (5). Since this date, one publication (30) reported higher rates of endometriosis detection in patients imaged on 3.0T systems versus those performed on 1.5T (LE4) while the absence of difference was recently reported in a multicentric cohort study (29) (LE3). As underlined by the SAR consensus (28) , no study compared the two systems in the same population. The 3.0T system offers an improved signal-to-noise (SNR) ratio, leading to high-spatial resolution images and precise depiction of DE locations. However, 3.0T systems exhibit increased image artifacts related to field heterogeneity, which can adversely affect fat-saturation techniques commonly used in endometriosis evaluations. The Dixon technique improves imaging quality by achieving better fat suppression. According to ESUR expert group, both 1.5T and 3.0T systems are valuable for evaluating DE (80%, 16/20)).

In line with the previous ESUR consensus based on literature research (LE 3) ESUR panelists confirmed the utility of phased arrays coils for DE evaluation at both 1.5T and 3T (95%, 19/20) due to a higher SNR than body coils. Finally, in line with the American and previous European consensus (5,28), all centers confirmed performing MRI with the patient in the supine position (100%, 20/20). In cases of claustrophobic patients, prone scanning could be advised.

Statement 9: There is no recommendation regarding for the use of a specific magnet strength, phased array coils are highly recommended at 1.5T and 3T and women should be placed in supine position (Grade C, strong agreement)

MRI Sequences and Contrast-Enhancement

T2-WI

As stated in the previous ESUR consensus (5), T2-weighted MR sequences without fat-suppression are essential for the evaluation of the female pelvis and are considered the best option for detecting DE (31) (LE2). All experts (20 out of 20 experts) recommend performing at least two different 2D T2W planes (sagittal and axial). The analysis of USL and parametria is improved by the acquisition of thin T2W oblique slices (LE3) (32,33) or 3DT2W acquisition with reformat in the dedicated plane.

T1-WI

As stated in the previous ESUR consensus (5), several studies have established T1W MR with or without FS as the gold standard for diagnosing endometriotic cysts (LE2) (34,35). T1W/T1W-FS or 3D Dixon technique is recommended by all experts (20/20) for evaluating adnexal endometriosis, superficial endometriosis, stromal inclusions in the fibroendometriosis plaque and differential diagnosis.

Diffusion-Weighted Imaging (DWI)

ESUR panelists strongly agreed that DWI is not systematically recommended for evaluating DE (80%, 18/20). However, when patient is referred for the characterization of an atypical endometrioma, DW sequence is recommended to apply O-RADS MR score (LE1) (18,36) and is also useful to diagnose seromucinous borderline or polypoid endometriosis (LE4) (19,37). Thus, DWI can be included in cases of unsupervised protocol studies or when differential diagnoses are being considered.

Intravenous Contrast-Enhanced MRI

ESUR panelists confirmed that the use of gadolinium is recommended only in selected cases (95%, 19/20) and strongly agreed in the following indications: atypical endometriomas, abdominal wall endometriosis, nerve locations.

Other sequences and recommendation

Systematic visualization of the kidneys and potential analysis of the right iliac fossa (i.e., cecum, appendix, small bowel) is recommended (95%, 19/20). Only 14 out of 20 experts systematically scan the upper abdomen up to the diaphragm, using T2W and T1W with and without FS and/or T1 DIXON. 3D-T2W imaging of the pelvis could be employed to obtain multiplanar imaging, reducing the acquisition time. Not enough evidence exist for a recommendation regarding the added value of susceptibility weighted imaging (SWI) which can improve the diagnostic accuracy of MRI by allowing the detection of hemorrhagic character of an endometriotic lesion (LE4) (38,39).

Finally, the ESUR expert consensus on technical recommendations for MRI technique in the evaluation of pelvic endometriosis highly recommended a clear and effective communication with the patient to alleviate patient anxiety, which can lead to an improvement in the quality of the examination (28)

Statement 10: MR protocol must at least include multiplanar T2W and T1W sequence and a sequence that visualizes the kidney (Grade A, Strong agreement). The following sequences may be optionally informative in specific situations: 2DT2W oblique thin sequence or 3DT2W (USL, parametrium) (Grade C, moderate agreement), DW sequence (atypical endometrioma) (Grade A, strong agreement), T1W after gadolinium (atypical endometriomas, abdominal wall endometriosis, nerve) (Grade A, strong agreement)

Supplemental material 3: Figures

S1. Superficial endometriotic peritoneal lesions in a 30-year-old woman. (a) Coronal T2-weighted MR image shows a small cystic lesion attached to the peritoneal surface of the torus uterinus (arrowhead). The adjacent proximal thirds of the uterosacral ligaments are slightly thickened (arrows). (b). Axial T1-weighted fat-saturated MR image shows a hyperintense signal (arrow) within the cyst, likely due to haemorrhagic content.

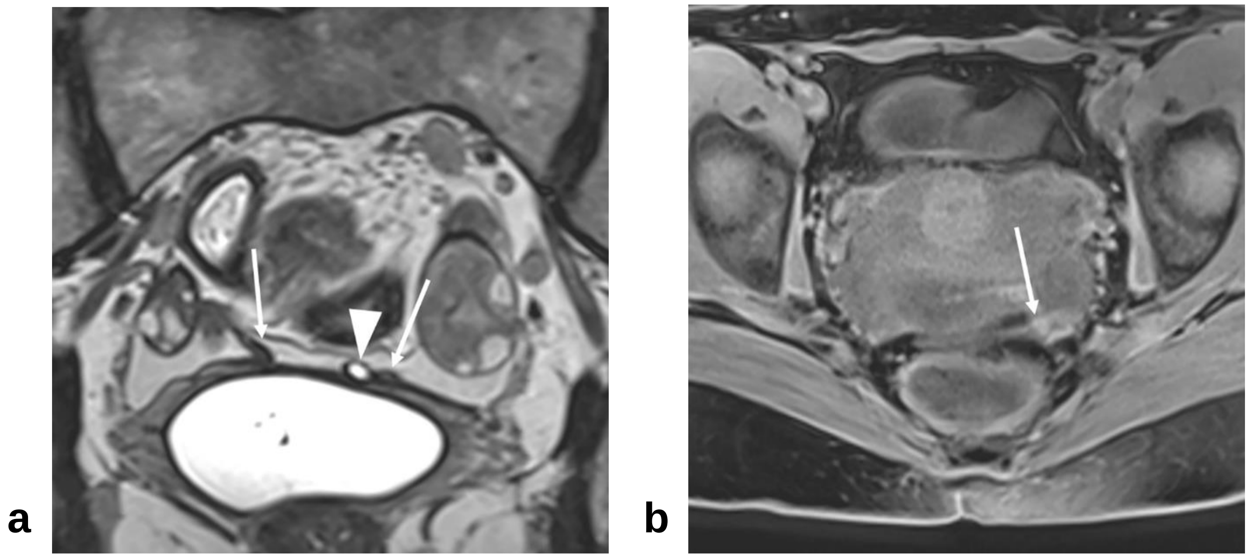

S2. Small left ovarian endometrioma in a 28-year-old woman. (a, b) Axial and sagittal T1-weighted fat-saturated MR images show a small ovarian cyst (< 1.0 cm) with high signal intensity, likely due to hemorrhagic content (arrows).

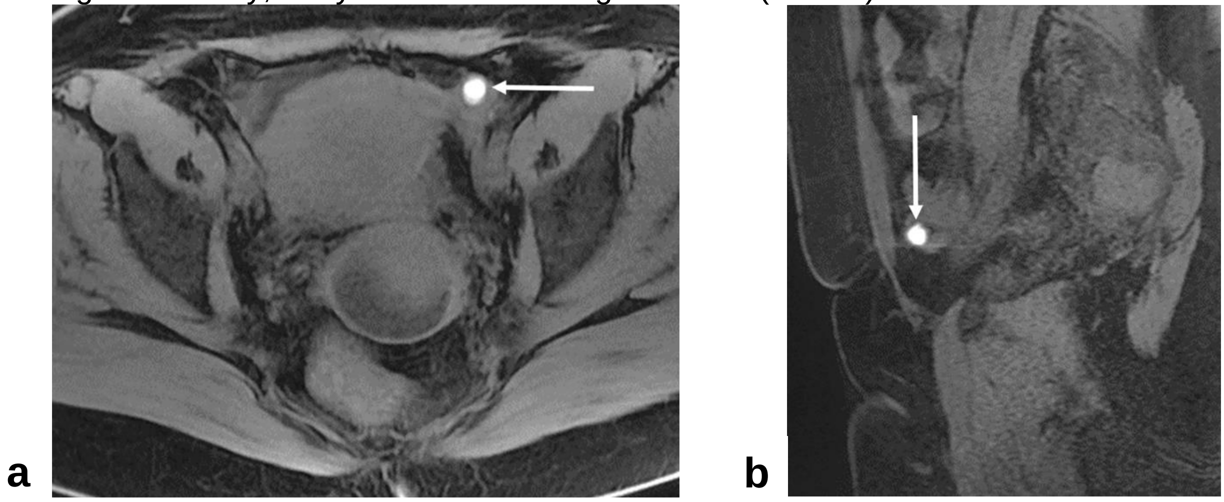

S3. Large ovarian endometrioma with heterogeneous content in a 31-year-old woman. (a) Axial T2-weighted MR image shows shading within the right ovarian endometrioma (black arrow) and a few T2 dark spots (white arrow) along the posterior cystic content, possibly corresponding to blood clots. (b) Axial T1-weighted fat-saturated post-contrast MR image with subtraction shows no solid enhancement within the cyst, reinforcing the avascular nature of the dark spots.

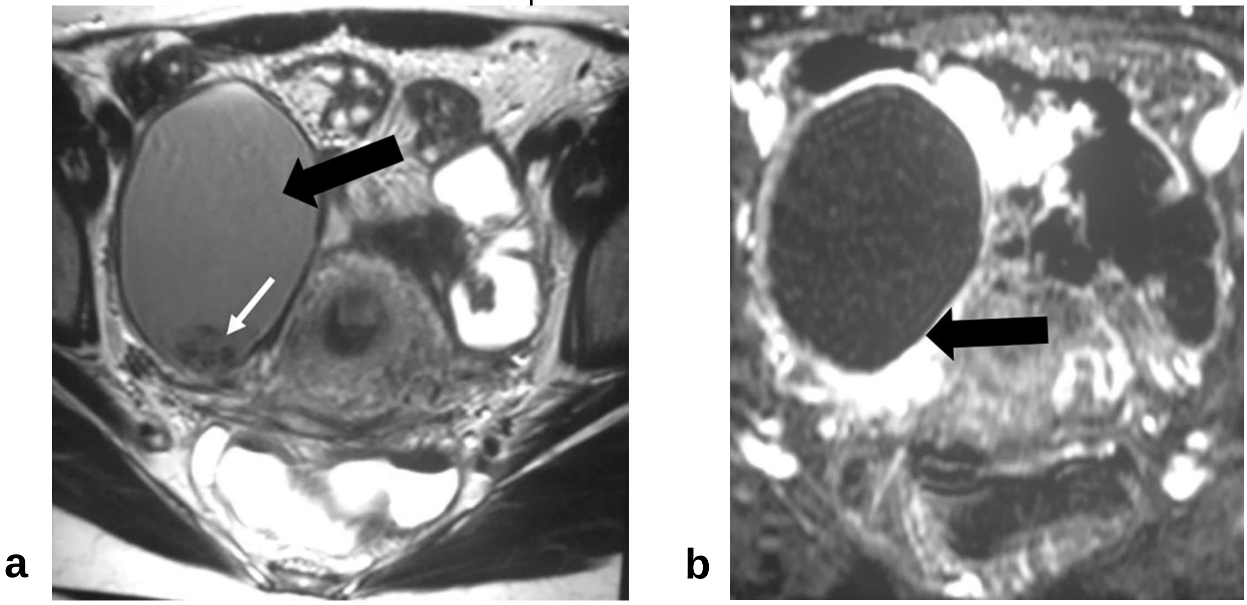

S4. Endometrioma and Ovarian clear cell carcinoma. The figure illustrates a typical left ovarian endometrioma, showing shading on axial T2-weighted (a) (*)and high signal on T1-weighted fat-saturated MR images (b) and no contrast enhancement on axial T1-weighted fat-saturated post-contrast MR image with subtraction (c) and dark on diffusion weigh image (DWI b1000) (d). The right ovarian cystic lesion shows shading on axial T2-weighted as well as left endometrioma but also enhanced papillary projection within the cyst with restriction on DWI (arrow), reinforcing the solid nature of atypical tissue within the cyst related to clear cell carcinoma arising from an endometrioma.

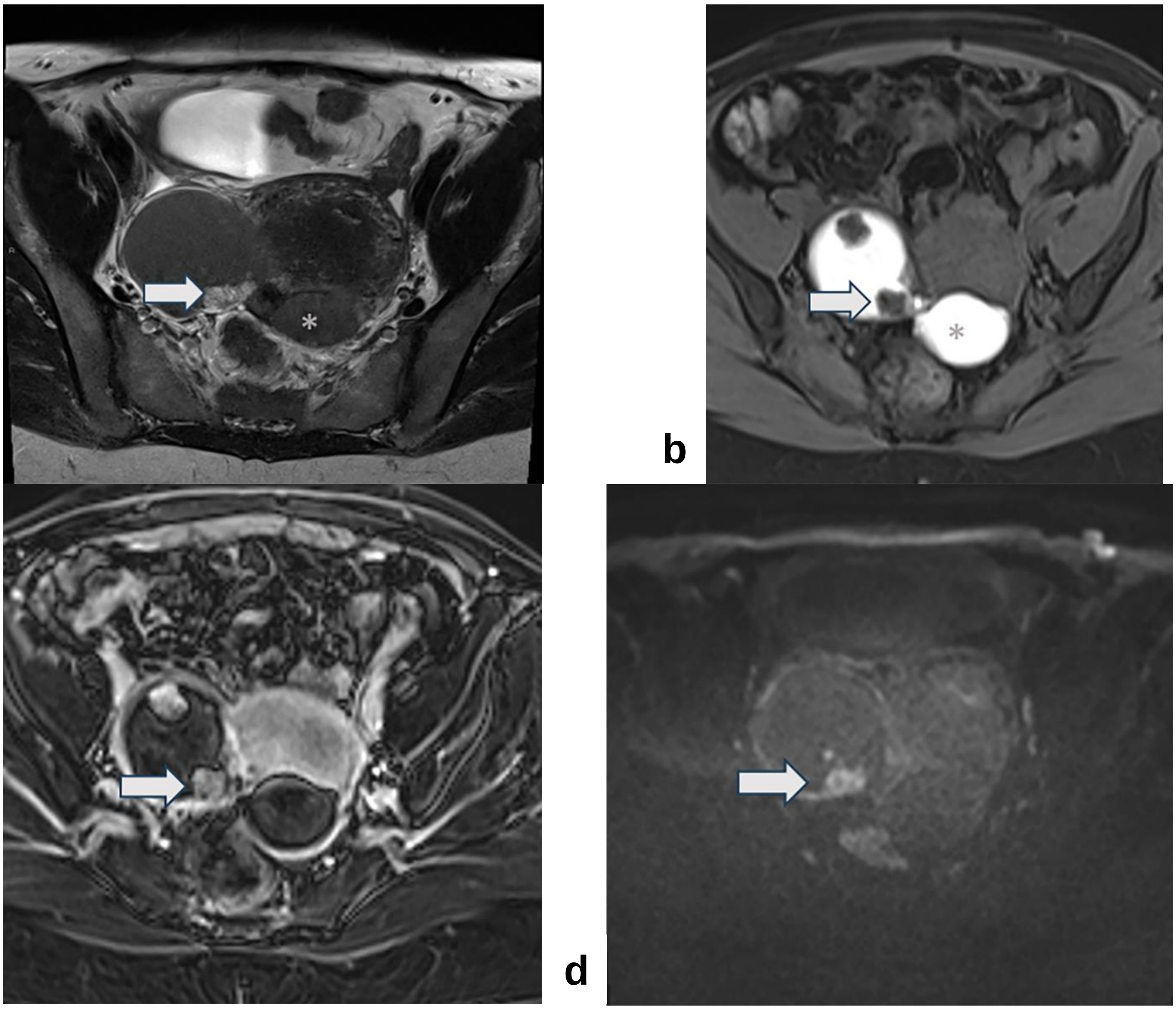
a b c d

S5. Left hematosalpinx associated with endometriosis in a 32-year-old woman. (a) Axial T2-weighted MR image shows shading within a tortuous tubular structure (arrows), suggestive of hematosalpinx. (b) Axial T1-weighted fat-saturated MR image clearly demonstrates the haemorrhagic nature of the tube's content (arrows), evidenced by the high signal intensity of the fluid. The left tube is medially displaced due to adhesions and anatomical distortion.

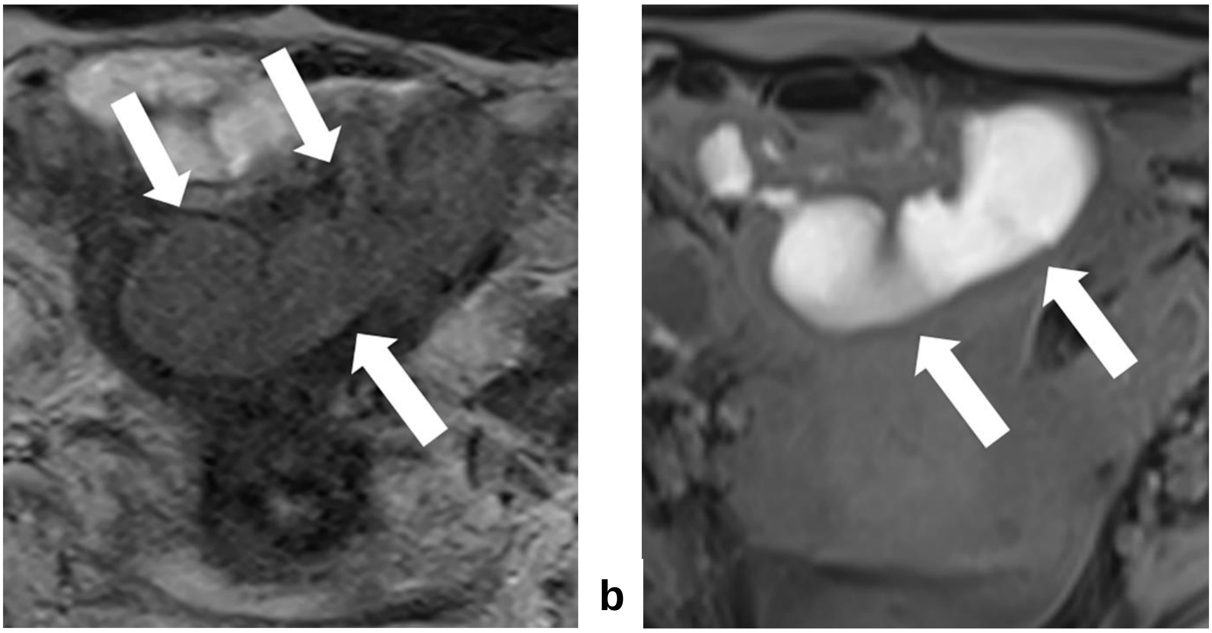

a b

S6. Different imaging examples of adenomyosis findings on sagittal T2-weighted MR images. (a) Mild diffuse adenomyosis in a 40-year-old woman. The MR image shows a mildly enlarged uterus with asymmetric thickening of the junctional zone (arrows) affecting the anterior and posterior walls, associated with small cystic cavities within the myometrium (arrowheads) representing the migrated glandular components. (b) Severe adenomyosis in a 44-year-old woman. The MR image shows a globular uterus with diffuse thickening of the junctional zone, compromising over 50% of the myometrial thickness (arrows), associated with cystic components, one of which contains a fluid-fluid level (arrowhead). (c) Adenomyoma in a 38-year-old woman. The MR image shows a low signal-intensity round mass with numerous cystic cavities in the anterior myometrium, characterized by poorly defined borders (circle).

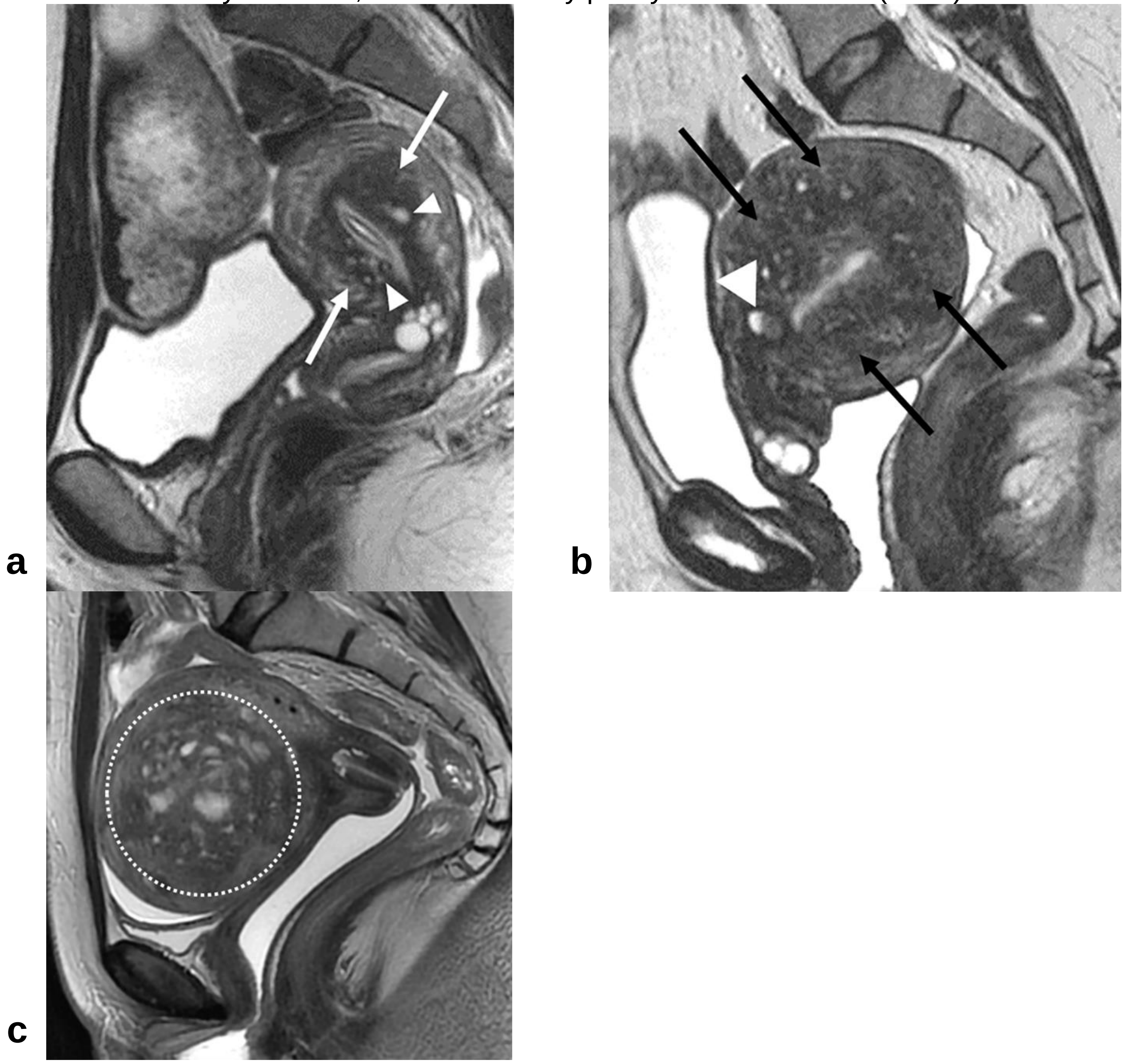

S7. Endometriosis of pouch of Douglas. Laparoscopic image (a) demonstrates extensive peritoneal adhesions with obliteration of the cul-de-sac (arrow). The rectal wall is adherent to cervix and torus uterinum. These aspects are correlated on axial T2-weighted MR image as complete obliteration of the pouch of Douglas with the presence of right lateral fluid collection (*), adhesions extending though uterus, rectum and medial displacement of both ovaries. UT= Uterus; O=Ovary; R=Rectum.

a

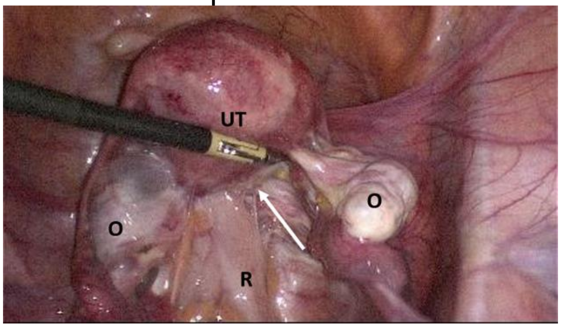

b

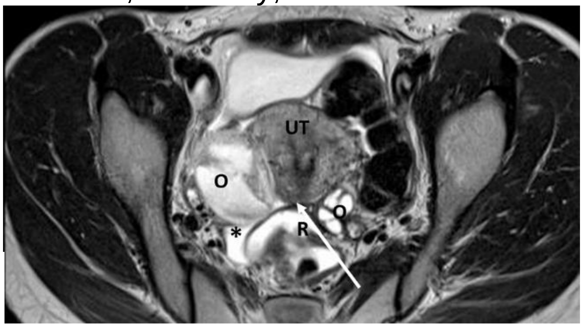

S8. Different examples of endometriosis infiltrating the myometrium, also known as external adenomyosis. (a) Axial T2-weighted MR image shows extensive endometriotic infiltration of the posterior uterine wall (the area inside the dashed line), associated with a rectal nodule (curved arrow) and a small left ovarian endometrioma (*). (b) Sagittal T2-weighted MR image demonstrates infiltration of the posterior uterine serosa and myometrium (the area inside the dashed line), associated with a retractile uterine retroflection and a bowel nodule (arrow). (c) Sagittal T2-weighted MR image shows infiltration of the anterior uterine wall (area inside the dashed line). Mild adenomyosis affecting the posterior uterine wall is also noted (arrow).

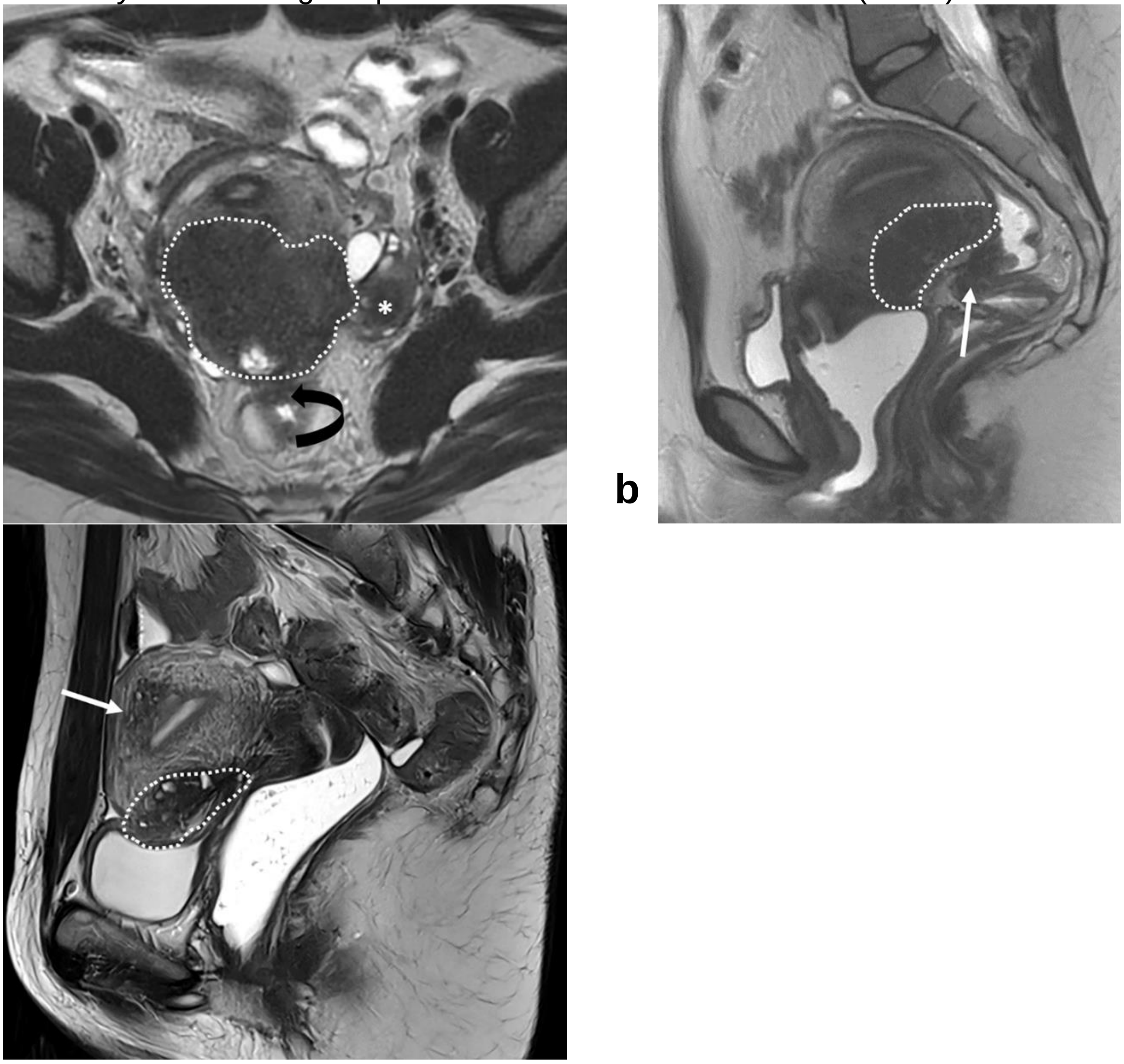

a b c

S9. Extensive left parametrial deep pelvic endometriosis with left ureteral stenosis in a 38-year-old woman. Axial T2-weighted images (a) shows a large heterogeneous endometriotic infiltrating mass at left parametrium (white arrow) occluding left ureter with upstream ureteral dilatation (black arrow) above the point of obstruction demonstrated on axial (b) and sagittal T2-weighted images (arrows) (c). Uro-RM illustrates the left hydronephrosis, ureteral and pyelocaliceal dilatation (arrowheads).

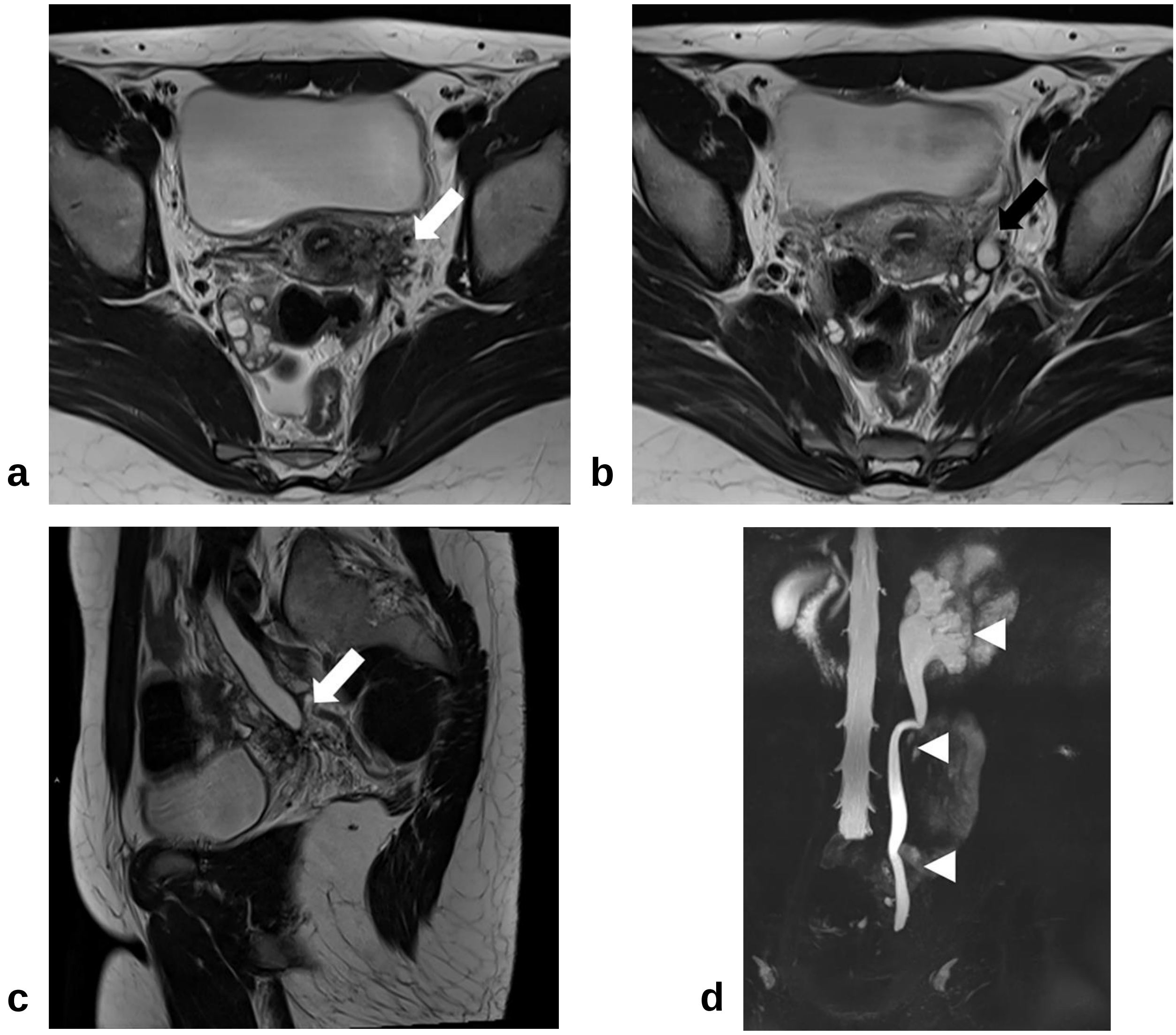

S10. Imaging examples of endometriosis affecting pelvic nerves. Images a, b and c, illustrates a patient with the right sciatic nerve completely effaced and encased by spiculated nodule with intermedium/low signal on axial T2-weighted MR image, centered at the sciatic notch anteriorly to the piriformis muscle (a) (white circle). This endometriotic nodule typically shows signal pattern of haemorragic microcysts with high signal on axial T1-weighted MR image and high contrast enhancement on axial T1-weighted fat-saturated post-contrast MR image with subtraction (black circle) (c). The images on d,e,f and g illustrates another patient with large right side wall deep endometriosis retractile mass in the obturator fossa (white circle) on T2-weighted MR image (d), affecting right obturator internus muscle leading to atrophy and hyperintensity (*) compared to left normal obturator internus muscle (curved arrow), on axial (d) and coronal T2-weighted MR image (e). The obturator nerve (black arrow) and pudendal nerve (white arrow) are encased by the nodule. The nodule infiltrated the right parametrium and caused dilatation of the right ureter (arrowhead) (e). The axial T1-weighted MR image (f) shows hyperintense microcysts in the nodule (white circle). The laparoscopic view (g) correlation of the referred endometriotic nodule (black circle). P: piriformis muscle.

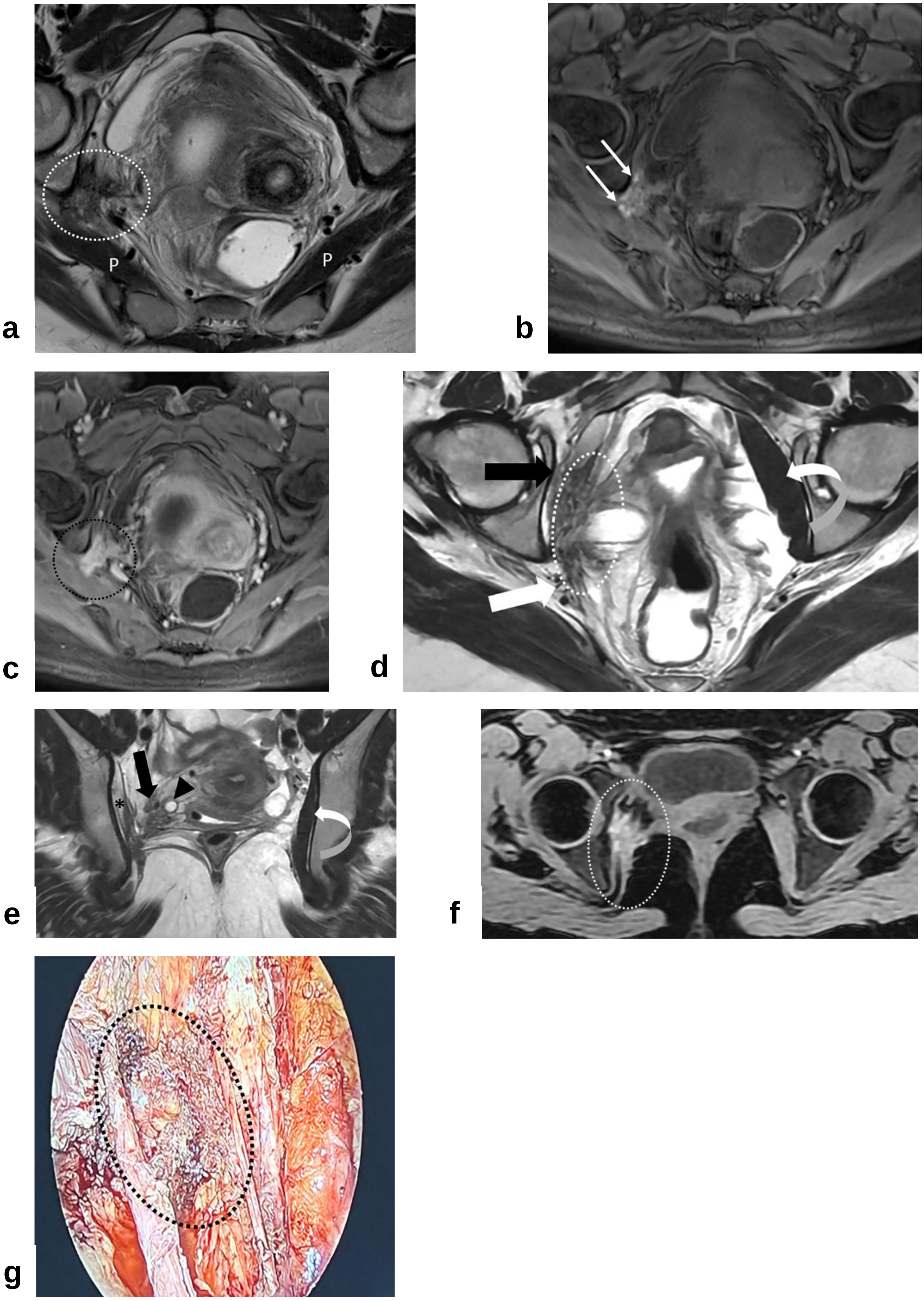
a
P
P
b
c
d
e
*
f
g
